# Initiation of voluntary movements at free will and ongoing 0.1-Hz BOLD oscillations in the insula—a pilot study

**DOI:** 10.3389/fnint.2014.00093

**Published:** 2014-12-08

**Authors:** Gert Pfurtscheller, Alexandre Andrade, Karl Koschutnig, Clemens Brunner, Fernando Lopes da Silva

**Affiliations:** ^1^Institute for Knowledge Discovery (BCI Lab), Graz University of TechnologyGraz, Austria; ^2^BioTechMedGraz, Austria; ^3^Institute of Biophysics and Biomedical Engineering, Faculty of Sciences, University of LisbonLisboa, Portugal; ^4^Institute of Psychology, University of GrazGraz, Austria; ^5^Center for NeuroScience, Swammerdam Institute for Life Sciences, University of AmsterdamAmsterdam, Netherlands

**Keywords:** 0.1-Hz oscillations, insula, voluntary movement, fMRI, BOLD

## Abstract

Recently we hypothesized that the intention to initiate a voluntary movement at free will may be related to the dynamics of hemodynamic variables, which may be supported by the intertwining of networks for the timing of voluntary movements and the control of cardiovascular variables in the insula. In the present study voluntary movements of 3 healthy subjects were analyzed using fMRI scans at 1.83-s intervals along with the time course of slow hemodynamic changes in sensorimotor networks. For the analyses of BOLD time courses the Wavelet transform coherence (WTC) and calculation of phase-locking values were used. Analyzed was the frequency band between 0.07 and 0.13 Hz in the supplementary motor area (SMA) and insula, two widely separated regions co-active in motor behavior. BOLD signals displayed slow fluctuations, concentrated around 0.1 Hz whereby the intrinsic oscillations in the insula preceded those in the SMA by 0.5–1 s. These preliminary results suggest that slow hemodynamic changes in SMA and insula may condition the initiation of a voluntary movement at free will.

## Introduction

How voluntary movements originate is a classic problem in Neuroscience. It is generally assumed that voluntary movements result from neuronal activity in premotor and motor cortical areas that precedes the conscious decision to move. Starting from analyzing the well-known Libet et al. (1983) experiment Brass and Haggard (2008) distinguished three aspects of a voluntary movement: “what” to move, “when” to move, and ultimately “whether” to move or not. Studies in which subjects freely choose between moving the right or left hand showed activation of medial frontal cortex. Brass and Haggard (2010) concluded that premotor and motor cortical areas generate action plans for a movement to occur, while the parietal cortex monitors how these plans are realized. In addition to the role of the medial frontal cortex with the executive motor area (SMA) and higher-order motor area (anterior cingulate cortex, ACC) there is also evidence that the anterior insular cortex (AIC) is involved. The AIC is not only a simple visceral sensory region but also responsible for body awareness and “gut feeling” (Craig, 2009). Both ACC and AIC are co-active in so many behaviors and also during cognition, pain and emotions (Yarkoni et al., 2011). Be as it may, it is noteworthy to underline that the AIC, particularly the rostral-frontal insular cortex (Cauda et al., 2011), besides its putative role in the initiation of movement, i.e., the “when” component, not only has a major role in the integration of visceral sensory information arising from baroreceptors and chemoreceptors within the cardiovascular system but also plays a role in the efferent control of baroreflex function (Verberne and Owens, 1998; Zhang et al., 1999; Craig, 2004). In this relationship it is important to note that one important feature of the baroreflex loop is its strong preference for oscillations around 0.1 Hz (Van Roon et al., 2004). Thus it appears that in the AIC different kinds of neuronal processes are intertwined: those associated with the initiation of voluntary movement, and those involved in the control of cardiovascular functions, although the precise neuronal networks supporting these intertwining processes are not yet clearly defined.

In general terms we may state that voluntary movements are internally driven. But what drives them internally? We hypothesized (Pfurtscheller et al., 2012a,b) that the intention to perform a voluntary movement at a given moment may be related to the dynamics of hemodynamic variables, which may be supported by the intertwined networks responsible for the timing of voluntary movements and for the control of cardiovascular variables, as described above. Preliminary results showed that the initiation of finger movements at free will, which occurs in about 40% of normal subjects studied at relatively constant intervals around 10 s, was time related to slow oscillations of autonomic blood pressure in the resting brain (Pfurtscheller et al., 2010). These oscillations tend to occur at frequencies around 0.1 Hz, the classic Mayer-waves (Mayer, 1876; Julien, 2006).

The goals of this pilot study are, first, to obtain further information about the role of slow hemodynamic oscillations in association with voluntary movement initiation and, second, to investigate more specifically time differences of slow intrinsic oscillations in two widely separated co-active brain areas prior to movement, the SMA and the insula. Furthermore this pilot study should help to clarify whether the popular time-frequency (t-f) tool, the Wavelet transform coherence (WTC), can be used to search for such phase differences in ongoing ~0.1-Hz oscillations and gives therewith references for the planning of a larger group-size study about voluntary movement at free will.

## Materials and methods

### Subjects and experimental paradigm

Three healthy male subjects aged 27, 30 and 35 years were studied. All were right-handed as determined by the Edinburgh Handedness Inventory. The experiments were in compliance with the World Medical Association Declaration of Helsinki. All subjects gave informed written consent before participating.

The subjects were requested to rest for 5 min with eyes closed, stay awake, and avoid any movements. After a short pause, a 10 min scanning period with self-paced voluntary brisk finger movements followed. Subjects were instructed to press a button at “free will” with the right index finger. No training was performed and no instruction was given about the timing of the movements. Data are reported only from the movement task.

### fMRI image acquisition and preprocessing

Functional images were acquired on a 3.0-T scanner (MagnetomSkyra, Siemens, 160 functional echo-planar images, voxel size 3.5 × 3.5 × 3.5 mm^3^, TR = 1830 ms, TE = 30 ms, FOV = 240 × 240 mm^2^). Functional data were pre-processed using the DPARSFA toolbox (Chao-Gan and Yu-Feng, 2010). The volumes were corrected for slice time and head motion. The data were normalized to MNI space using the EPI template provided by SPM, resampled to 3 × 3 × 3 mm^3^ isotropic voxels, and smoothed with a 4 mm FWHM Gaussian kernel. Finally, time courses of 90 regions based on the AAL atlas (Tzourio-Mazoyer et al., 2002) were extracted and signals analysed in left SMA and left insula. In this pilot study we used the AAL atlas based on neuroanatomy and did not make a detailed differentiation of the insula in specific subregions with high spatial resolution (Kelly et al., 2012) and thus used simply more global descriptors, because we focused on slow ongoing activity oscillations in widely separated anatomical areas.

### Wavelet transform coherence analyses

Wavelet transform coherence (WTC) is a time- and frequency-specific measure of association between signals that has been applied to brain functional data several times in the past (Klein et al., 2006). Although WTC, being a t-f approach, benefits from high temporal sampling rates such as the ones achieved with electroencephalography, its ability to achieve temporal and spectral specificity in the case of a much slower signal such as fMRI time-series has been demonstrated (Müller et al., 2004; Chang and Glover, 2010). WTC was chosen because it combines the following desirable features: (i) Instances of previous application in the context of fMRI; (ii) Solid theoretical foundations; (iii) Straightforward implementation; and (iv) proper handling of t-f resolution in the sense that temporal resolution increases with frequency while spectral resolution decreases. WTC was applied to the time series of the selected ROI pair using the “Cross wavelet and wavelet coherence” toolbox implemented in Matlab^TM^ (Grinsted et al., 2004). The Morlet wavelet was chosen as mother wavelet. Magnitude and phase components of the resulting t-f maps were extracted. The phase component was used to compute the Phase-Locking Value PLV (Lachaux et al., 1999), which basically reflects stability of the phase difference between two signals across a sliding time window. This computation was performed for every frequency and for every time point, with a window size of 5 cycles.

In this pilot study we used the Rayleigh test (Berens, 2009) to test whether phase differences between insula and SMA BOLD signals are uniformly spread across the trigonometric circle or if they are concentrated in specific segments of the circle. In the case of a significant *p*-value, the hypothesis of a uniform distribution can be rejected, due to a clustering of the phase differences in one or more segments. *P*-value plots as a function of frequency can be drawn to show to what extent frequencies in the range of interest (0.07–0.13 Hz) are associated with low *p*-values. A “dip” in the *p*-value plot is indicative of frequency-specific non-randomness of phase difference distribution. The computation of the circular histogram allows visualizing the distribution of phase difference values across the time series as well as pinpointing the location of the segment with the largest clustering density.

### Hemodynamic responses

For response calculation the BOLD time courses were linearly detrended for each ROI and resampled from 0.5465 Hz to 10 Hz. The resampling procedure assumes that each BOLD wave is represented by at least 2 samples and no aliasing occurs. In this way oscillations with frequencies lower than the Nyquist frequency of 0.273 Hz can be displayed correctly. Movement onset was used as the trigger. For each response also the trial-to-trial variance was calculated (the standard error, SE, was displayed) and the positive peak after movement was determined. We used paired *t*-tests to find out whether the positive peak after the button press was significantly different from the average BOLD activity during the baseline period taken from −6 s to −0.5 s before the movement, and whether the latency differences of the selected ROI pairs were significantly different. We conducted individual *t*-tests for each subject/ROI combination and used Bonferroni correction to control the family-wise error rate.

## Results

### Single trial phase differences

**Figure 1:**
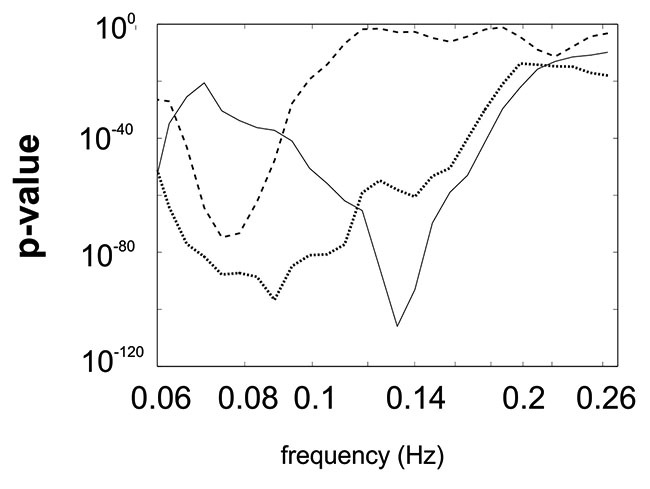
**Superimposed *p*-value plots for the frequency range between 0.06 Hz and Nyquist frequency for the 3 subjects (S1 full line, S2 dotted line, S3 stippled line)**.

**Figure 2:**
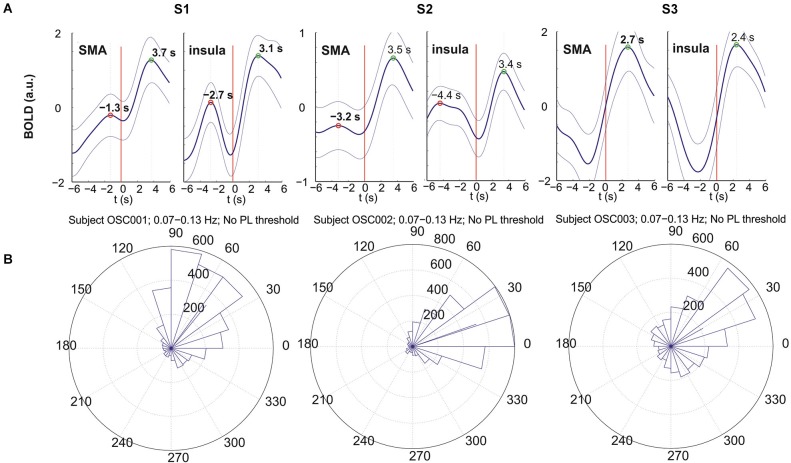
**BOLD responses (A) and circular histograms (B) for subjects S1 (left), S2 (middle) and S3 (right)**. **(A)** Movement-evoked BOLD responses from left insula (right panel) and left SMA (left panel); the thick line indicates the mean and the 2 thin lines the corresponding ± SE; button press is indicated by the red vertical line. **(B)** Circular histograms of the phase differences between insula and SMA calculated for the frequency band 0.07–0.13 Hz. This histogram shows the distribution of phase difference values around the trigonometric circle from 0 to 360°. The side length of each triangle reflects the amount of phase values contained within the angle interval enclosed by the triangle. The location of the segments between 0° and ~90° indicates that the oscillations in the insula are leading.

All *p*-values of the Rayleigh test were highly significant, and therefore the null hypothesis of uniformly distributed phase differences was rejected throughout. Remarkable is that frequencies around 0.1 Hz correspond to a “dip” in the *p*-value plot, meaning that this frequency (“dip” frequency) shows the lowest random phase difference distribution within the 0.07–0.13 Hz band (see Table 1 and Figure 1). The most pronounced dip was found at 0.13 Hz in S1 while the dip was relatively broad in S3 with a minimum at 0.07 Hz and very broad in S2 with a dip at 0.09 Hz. This indicates that in S1 one small frequency band displayed a phase coupling while in S2 and S3 the band was broader. Circular histograms (showing the distribution of phase difference values, ranging from 0 to 360°, around the trigonometric circle) are displayed in Figure 2B and the mean time differences (in seconds) between left insula and left SMA are indicated in Table 1. The 0.1 Hz oscillations in the insula preceded those in the SMA by 0.5–1 s.

**Table 1:** **“Dip” frequencies obtained by the Rayleigh-test in the range 0.06—Nyquist frequency and corresponding mean time delays in seconds between left insula and left SMA**.

	Frequency (Hz) of “dip” in Rayleigh p-value plot	Mean time delay (s)
S1	0.13	1.02
S2	0.09	0.45
S3	0.07	0.57

### Averaged responses

Each subject initiated voluntary movements, but displayed different patterns. Namely, subject S1 made 28 finger movements at relatively random intervals with a mean interval of 19.2 s (SD = 8.3 s), S2 performed 78 voluntary movements at relatively regular intervals of 8.1 s (SD = 2.9) and S3 made only 19 movements with a mean interval of 30.2 s (SD = 13.5 s). The averaged evoked BOLD responses (±SE) following button press were studied within the left SMA and left insula (Figure 2A). The large SE indicates a pronounced trial-to-trial variability in each subject. The right part of the curves from 0 (movement-onset) to 6 s post-movement represents a reliable response with peak latencies (indicated in Figure 2A) between 2.4 s and 3.7 s. The results show that all positive peaks are significantly different from the preceding baseline activity (*p* < 0.05) except for subject S2 (insula). The evoked hemodynamic responses in the left SMA and left insula revealed peak latency differences of 0.6 s (S1: 3.7 s − 3.1 s), 0.1 s (S2: 3.5 s − 3.4 s) and 0.3 s (S3: 2.7 s − 2.4 s) with insula leading (Figure 2A), but only the difference of S1 was significant (*p* < 0.02).

## Discussion

The different movement patterns in the subjects studied (19, 28 and 78 movements, respectively, in 10 min) give evidence that the subjects pressed the button at free will, but also show that in the case of a small number of movements there is still present ongoing intrinsic activity. In this relationship we refer to the findings of Monto et al. (2008) that the slow ongoing intrinsic oscillations reflect excitability changes in cortical networks correlated with slow fluctuations in human psychophysical performance. Thus a movement may be initiated if such an excitability increase passes over a given threshold. Based on this assumption and the different number of movements we propose that in studies of the dynamics of voluntary movements occurring without any instruction about timing, one should consider these movements in the context of two different brain states: namely the resting state and the active state.

In the resting state ongoing intrinsic oscillations around 0.1 Hz were observed in the 2 selected ROIs, the left SMA and the left insula, however, with different dominant frequencies in different subjects and a time difference of ~ 1 s with insula leading. The frequencies (dip frequencies ) were 0.13 Hz in S1, 0.09 Hz in S2 and 0.07 Hz in S3 (see Table 1). This supports not only findings about the existence of ongoing intrinsic oscillations around 0.1 Hz in human oxygen availability, ECoG and EEG signals (Cooper et al., 1966; Pfurtscheller, 1976; Vanhatalo et al., 2004; Foster and Parvizi, 2012; Pfurtscheller et al., 2012b) but also shows that the activity is spreading from the insula to higher-order motor areas. Remarkably, Fox et al. (2007) documented that the ongoing intrinsic neuronal driven fluctuations can have an impact on the spontaneous button press force and induce BOLD changes 2–3 s later. Such a relationship between slow ongoing intrinsic BOLD fluctuations and behavior was named “BOLD-behavior effect”. Further investigations are necessary to check not only whether the “BOLD-behavior effect” plays a role in the initiation of freely-voluntary movements during slow ongoing BOLD oscillations but also about the role of the Mayer-waves. Noteworthy is the finding of a former study (Pfurtscheller et al., 2010) that some subjects initiated voluntary movements frequently at the decreasing slope of the slow blood pressure wave (Mayer-wave).

The active state comprises the preparation and execution of a brisk movement, which is associated with the processing of reafferent input from kinesthetic receptors evoked by the movement itself (Deecke et al., 1969). The movement-evoked BOLD response represents neuronal activation that takes 2–3 s to develop (neurovascular coupling) and peaks in the somatomotor cortex approximately 5–6 s after movement onset (Cunnington et al., 2005; Steinbrink et al., 2006; Fox et al., 2007). If we take into account the neuronal activation in the SMA approximately 2 s prior to the self-initiated movement (Deecke et al., 1969; Ball et al., 1999; Cunnington et al., 2005) the related BOLD response peak is expected at ~ 3–4 s after movement onset. This is in agreement with our results with BOLD peak latencies in the SMA between 2.7 s and 3.7 s (see Figure 2A).

The BOLD responses displayed an oscillatory behavior in all 3 subjects most pronounced in the insula. The period of these responses (time difference between positive post movement and pre movement peaks; see Figure 2A) was the shortest in S1 (~6 s) and the longest in S3 (estimated ~12 s). Interestingly these periods correspond approximately to the periods of the intrinsic oscillations in S1 (~7 s) and S3 (~14 s). This is first evidence that in these subjects the timing of movement-onset was approximately time-locked to the intrinsic activity.

In S2 the period of the oscillatory response was ~ 8 s (see Figure 2A) and corresponding to the mean inter-movement interval of 8.1 s while the intrinsic oscillations had a period of ~ 11 s (dip frequency 0.09 Hz). We can speculate therefore that S2 initiated the voluntary movements at relatively regular intervals of ~ 8 s most likely time-locked to each second respiratory cycle (corresponding to a respiration frequency of ~ 0.25 Hz). Such a respiration-related initiation of voluntary movements is not uncommon and has been documented recently (Pfurtscheller et al., 2010). The direct coupling between respiratory and cardiovascular centers in the brainstem and the existence of a respiration-related stimulation of arterial baroreceptors, cardiopulmonary receptors and stretch receptors in the chest wall (Saul et al., 1991) may contribute to such behavior. The BOLD response latency difference between insula and SMA in S2 was small and not significant, meaning that the responses were about concomitant in insula and SMA which lead to suggest also a respiration-related movement initiation.

The pilot study suggests that the WTC method is suitable to investigate the dominant frequency of slow oscillations in different areas and provide information about phase (time) shifts. However the AAL atlas used did not have the power to delineate precisely the subdivisions of the insular region. Nonetheless we measured the activity of the insular region globally, which may be expected to reflect, at least partially, the activity of AIC, although the insula is not a homogenous structure. We should emphasize that in this paper our primary aim is to report the first results of this approach in a most economical way; this may be considered as the presentation of a proof-of-principle, in which the main findings are put forward. A more extensive and comprehensive study is in preparation.

Given the small amount of data (only 3 subjects and 10 min of data in each subject), novel results are obtained but it is hard to draw valid conclusions. For a large size study the following improvements are recommended: (i) use a short scanning rate (TR < 1 s) to avoid respiration-related aliasing; (ii) record pulse or heart rate within the scanner to check whether the blood pressure waves (Mayer waves) play any role in initiation of movement; (iii) record the breathing cycle to monitor respiration-related movement initiation; (iv) study more pairs of ROIs within the motor and limbic systems to obtain reliable estimates of time differences; (v) investigate phase locking not only during movement but also in the resting state; and (vi) study distinct subregions of the insula and not the insula as a single region.

## Conflict of interest statement

The authors declare that the research was conducted in the absence of any commercial or financial relationships that could be construed as a potential conflict of interest.
